# Blossom-end rot: a century-old problem in tomato (*Solanum lycopersicum* L.) and other vegetables

**DOI:** 10.1186/s43897-021-00022-9

**Published:** 2022-01-12

**Authors:** Yasin Topcu, Savithri U. Nambeesan, Esther van der Knaap

**Affiliations:** 1 Institute of Plant Breeding, Genetics and Genomics, University of Georgia, Athens, GA 30602 USA; 2 Department of Horticulture, University of Georgia, Athens, GA 30602 USA

**Keywords:** Blossom-end rot (BER), Reactive Oxygen Species (ROS), Ca^2+^ deficiency, Abiotic stress, Cell wall, Tomato, Antioxidants, Plant growth regulators, Fruit morphology

## Abstract

Blossom-end rot (BER) is a devastating physiological disorder affecting vegetable production worldwide. Extensive research into the physiological aspects of the disorder has demonstrated that the underlying causes of BER are associated with perturbed calcium (Ca^2+^) homeostasis and irregular watering conditions in predominantly cultivated accessions. Further, Reactive Oxygen Species (ROS) are critical players in BER development which, combined with unbalanced Ca^2+^ concentrations, greatly affect the severity of the disorder. The availability of a high-quality reference tomato genome as well as the whole genome resequencing of many accessions has recently permitted the genetic dissection of BER in segregating populations derived from crosses between cultivated tomato accessions. This has led to the identification of five loci contributing to BER from several studies. The eventual cloning of the genes contributing to BER would result in a deeper understanding of the molecular bases of the disorder. This will undoubtedly create crop improvement strategies for tomato as well as many other vegetables that suffer from BER.

## Introduction

Vegetable production is challenged by a range of biotic and abiotic factors, often resulting in a substantial loss of the produce in each growing cycle. As the population is growing, the world is facing increasing demands for a stable food supply grown on agricultural lands across the globe. Unfortunately, abiotic stresses are becoming increasingly more prevalent especially in light of climate change. Climate-related changes, which are exemplified by extreme air and water temperature, increased frequency and intensity of rainfall, intense hurricanes and so forth are becoming more prevalent (Wuebbles et al. 2017; Hoegh-Guldberg et al. 2018; U.S. Environmental Protection Agency 2021). Undoubtfully, these extreme weather events will lead to an increase in stress related diseases and disorders (U.S. Global Change Research Program 2009). Short, and long-term impacts of climate change are expected to further increase these extreme weather conditions; thus, the stability of food supplies and crop productivity will continue to be affected adversely from these extreme events (Motha and Baier 2005; U.S. Global Change Research Program 2009; Lobell et al. 2013; Hoegh-Guldberg et al. 2018).

**Fig. 1:**
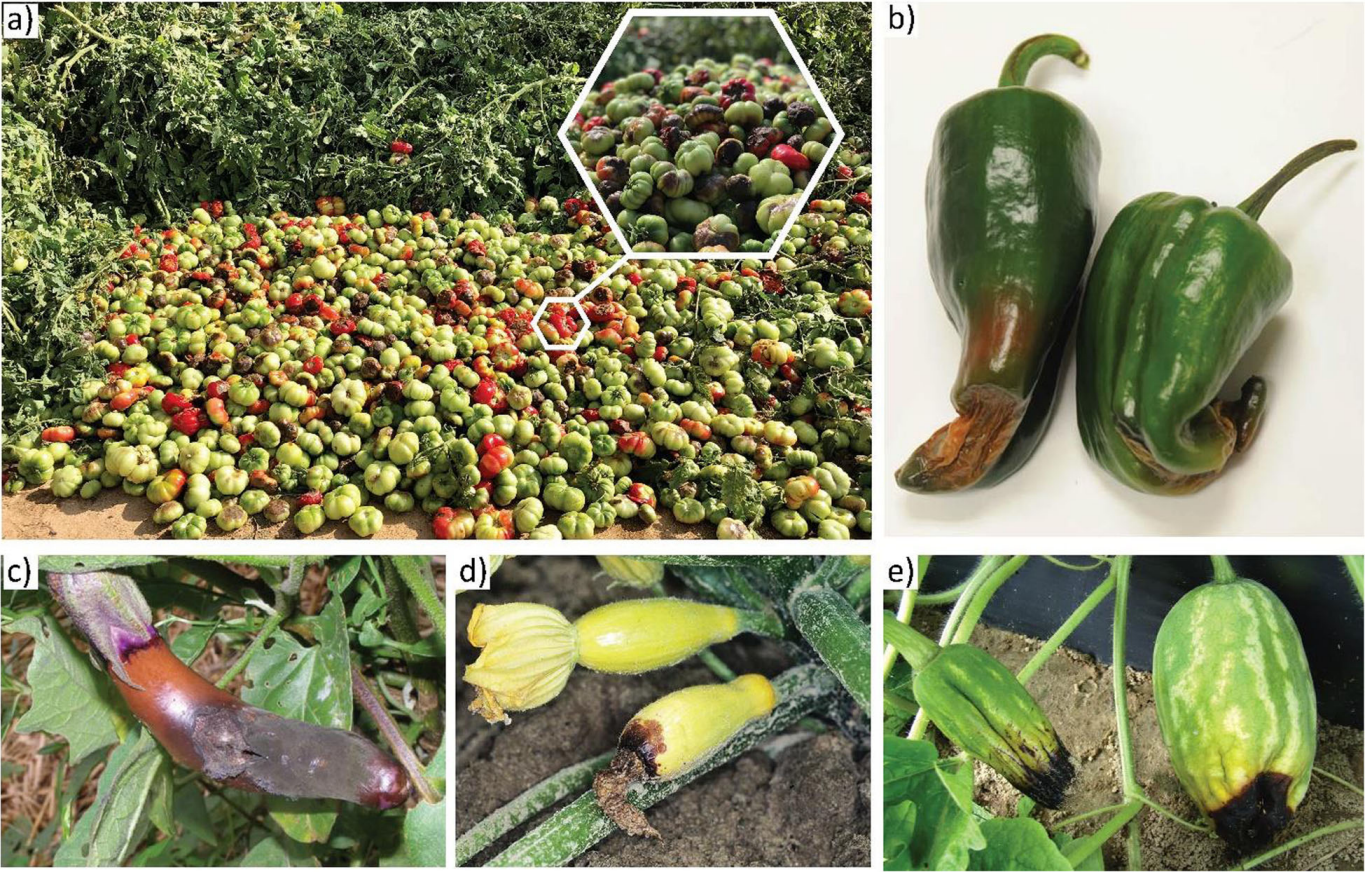
Blossom-end rot in various fruit and vegetable crops. **a**) BER in tomato. **b**) BER in pepper, image credit (WSU NWREC, 2021). **c**) BER in eggplant, image credit (University of Minnesota Extension, 2021). **d**) BER in squash, image credit (Voyle, 2021). **e**) BER in watermelon, image credit (UF/IFAS, 2021)

BER is one of the most devastating physiological disorders that affect various crops such as tomato (*Solanum lycopersicum* L.), pepper (*Capsicum annuum* L.), watermelon *(Citrullus lanatus* (Thunb.) and eggplant (*Solanum melongena* L.) (Taylor and Locascio 2004; Díaz-Pérez and Hook 2017) (Fig. 1). A related disorder in apple is bitter pit (Bangerth 1979; de Freitas et al. 2010), BER and related disorders affect mostly the fruit, but other organs such as leaves, and flowers can suffer as well. Various leafy vegetables suffer tipburn (Kuo et al. 1981; Francois et al. 1991; Barta and Tibbitts 2000; Macias-González et al. 2019; Su et al. 2019) and other vegetables such as celery and cauliflower are affected by disorders that appear similar to BER (Geraldson 1952; Rosen 1990; Bouzo et al. 2007; Bianco et al. 2015). Combined, these physiological disorders can lead to significant yield losses especially in subsistence and organic farming (Ikeda and Kanayama 2015; Hagassou et al. 2019). As the demand for organic produce is increasing, the impact of abiotic stresses on this sector may become substantial as well. As an example, Hickory Hill Farm in Carlton GA, USA faced a challenging season in 2018 when they lost almost 80% of the organically grown tomatoes to BER (Josh Johns and Gary Shaw, personal communication). BER was first described in tomato more than 120 years ago as a physiological disorder caused by inconsistent watering (Selby 1896), a notion that has held up until today. The early studies also indicate that BER is of great concern as it was linked to significant crop losses caused by canopy transpiration rate and the use of ammonium-based fertilization (Stuckey 1916; Wedgworth et al. 1927; Chamberlain 1933).

In this review, we summarize the recent findings on the development of BER from research primarily conducted in tomato. These findings are starting to shed light on the molecular basis of the onset of BER as well as crop improvement strategies that can be applied in the near future.

### Development of BER symptoms

**Fig. 2:**
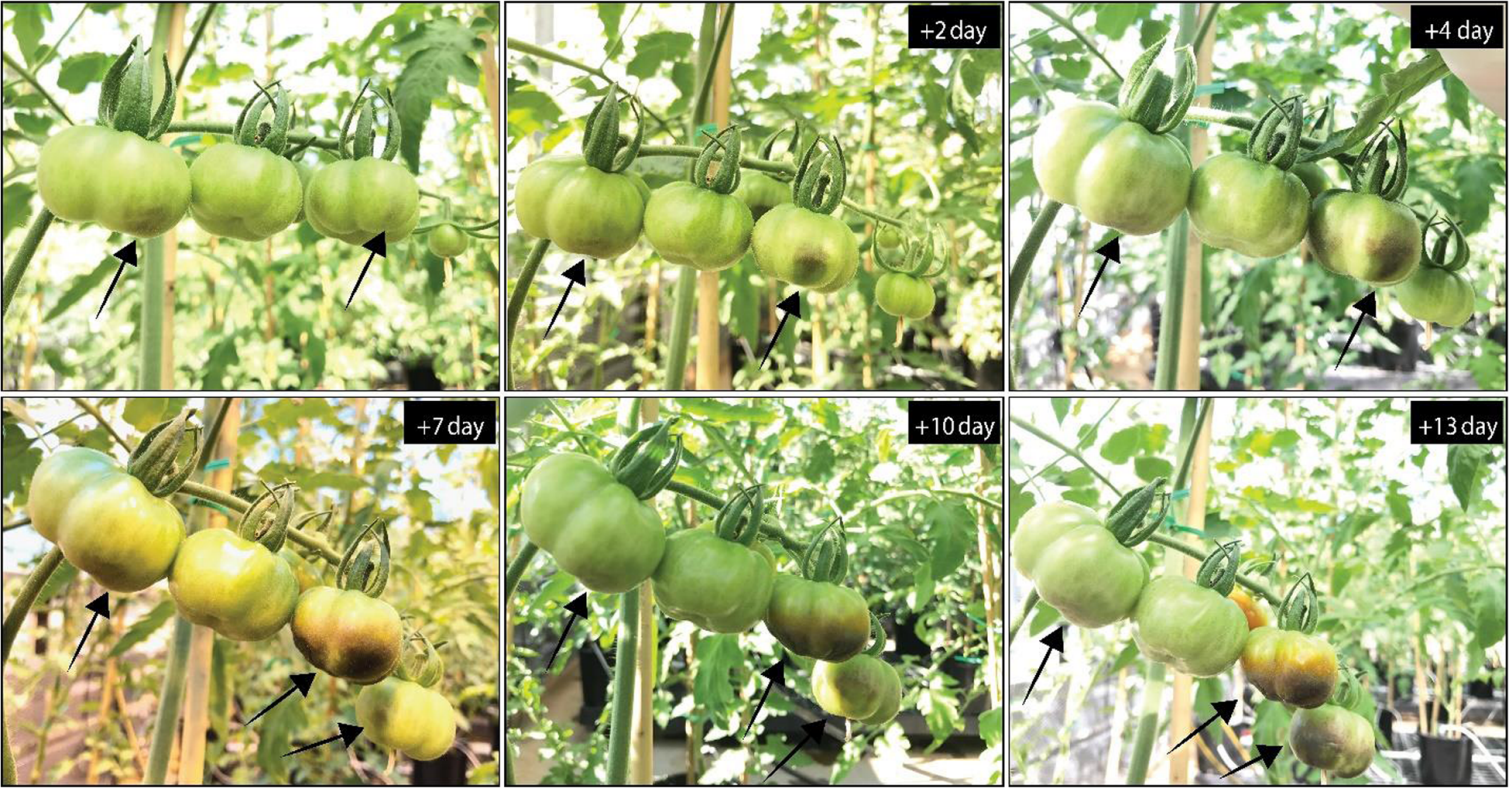
BER development in four fruits on one inflorescence. After first BER appearance was observed (top left panel), subsequent images were taken at time intervals in days from the initial image. Arrows indicate BER afflicted fruits. The BER on the first fruit did not expand to the entire fruit whereas the last fruit is consumed by BER in a short period of time

The initial external symptoms of BER in tomato are often observed on the distal portion of the fruit during the second week after pollination but can also occur later during development at five weeks after pollination (Spurr 1959; Marcelis and Ho 1999; Saure 2001; Ho and White 2005; de Freitas et al. 2018; Rached et al. 2018). Typical symptoms appear as small light colored, water soaked spots on the blossom end of the fruit which is associated with cell plasmolysis and leaky membranes (Ho and White 2005) (Fig. 2). BER symptoms usually appear externally on the pericarp at the distal end, but affected areas may also occur in the internal distal placenta tissue without visible external symptoms (Brust 2004; Hochmuth and Hochmuth 2009). After BER induction, BER-affected areas often expand and turn into brown necrotic regions covering a significant proportion of the fruit and in some extreme cases affect the entire fruit. Occasionally, BER fails to expand, and the afflicted areas disappear. The symptoms can be exacerbated if they occur soon after pollination and, in such cases, the fruit never attains its maximum size. BER-afflicted areas often become prone to invasion from secondary pathogens such as saprophytic *Alternaria* fungal species (Brust 2004; Hochmuth and Hochmuth 2009).

### Relationship between Ca^2+^ and BER

Findings from many studies have suggested that Ca^2+^ deficiency initiates BER incidence (Shear 1975; Adams and Ho 1993; Taylor and Locascio 2004; de Freitas et al. 2012b; Watanabe et al. 2021). During fruit growth, the differential Ca^2+^ concentrations between the proximal (high) and distal (low) end of the fruit is correlated to the appearance of BER such that the higher the difference, the higher incidence of BER (Franco et al. 1994). Ca^2+^ plays an essential role in plant growth and development where it fulfills three main functions. Ca^2+^ acts a secondary messenger (Kudla et al. 2010; Thor 2019), and the subcellular concentration in the cytosol, vacuole and apoplast are tightly regulated by Ca^2+^-ATPases, H^+^/Ca^2+^ exchangers, and channel proteins at different cellular membranes (Clarkson et al. 1993; Clapham 2007; Kudla et al. 2010; Thor 2019). Second, Ca^2+^ has a structural role in determining the rigidity of the cell wall through cross-linking with the de-esterified pectin in the middle lamella (Micheli 2001; Hepler and Winship 2010; Thor 2019). The largest Ca^2+^ pool of at least 60% is localized to the cell wall (Demarty et al. 1984). And third, free apoplastic Ca^2+^ concentration maintains the cell membrane integrity through connecting the phospholipids and proteins at the plasma membrane (Hepler and Winship 2010; Marschner 2011; Thor 2019). Ca^2+^ in BER development is associated with the aberrant regulation of its partitioning and distribution in different cellular compartments. For instance, apoplastic Ca^2+^ concentration specifically in the distal end of the fruit, rather than total Ca^2+^ concentration in the distal part, are negatively correlated to BER development (Ho and White 2005; de Freitas et al. 2011b). Ca^2+^ homeostasis can be perturbed by expression of Arabidopsis *sCAX1* (*Cation Exchanger 1*), encoding a functional Ca^2+^/H^+^ antiporter in tomato. *sCAX1* encodes a N-terminal truncated version of the full-length gene that does not contain its regulatory region and therefore is constitutively active. When *sCAX1* is expressed in tomato, 100% of the fruit exhibited BER symptoms (Park et al. 2005; de Freitas et al. 2011b). The *sCAX1* tomato exhibited higher total water soluble and fruit Ca^2+^ concentrations compared with the control. However, *sCAX1*-expressing tomato plants increased the transport of Ca^2+^ from the cytosol to the vacuole resulting in lower cytosol and apoplast Ca^2+^ concentration compared to non-transformed control. These results support the notion that altered Ca^2+^ homeostasis among different cellular compartments interferes with the signaling cascade that orchestrates the induction of downstream responses to BER or prevent BER from happening altogether (de Freitas et al. 2011b). The altered Ca^2+^ distribution is proposed to disrupt the integrity and function of the cellular membranes, which in turn could lead to leakage of solutes into the extracellular space resulting in BER (Ho and White 2005; Park et al. 2005; de Freitas et al. 2011b).

The majority of the cell wall Ca^2+^ is bound to the de-esterified pectin whereas the remainder is in free form (Marschner 2011). Pectin is the major component of the middle lamellae in plants (Demarty et al. 1984; White and Broadley 2003; Marschner 2011) and is synthesized in the Golgi apparatus to be secreted into the cell wall in a highly methylesterified form (Goldberg et al. 1996; Micheli 2001; Wormit and Usadel 2018). During growth, the secreted pectin undergoes modifications by pectin methylesterases (PMEs) which is countered by pectin methylesterase inhibitors (Micheli 2001; Bosch et al. 2005; Pelloux et al. 2007; Palin and Geitmann 2012; Wormit and Usadel 2018). Ca^2+^ interacts electrostatically with the negatively charged carboxyl groups on the demethylated pectin facilitating the cross linking of the pectin molecules and stiffening of the cell wall (Micheli 2001; Wormit and Usadel 2018). Retaining the concentration of freely available apoplastic Ca^2+^ is critical to maintain membrane stability and for cellular responses to BER. The concentration of free apoplastic Ca^2+^ is dependent on pectin bound Ca^2+^ which is required for cell wall stability. Thus, when cell wall and membrane stability collapses, BER symptoms can be initiated in response to the stress (de Freitas et al. 2011b; Marschner 2011; Watanabe et al. 2021).

The suspected role of pectin in sequestering Ca^2+^ and causing BER has led to studies that aimed at modifying pectin properties. Using gene silencing, antisense expression of pectin methylesterase *LePME3* (*Solyc07g064190*) increased water-soluble *Ca*^*2+*^ concentration in tomato fruits resulting in less electrolyte leakage and less BER (de Freitas et al. 2012b). Note however, that the antisense expression led to the downregulation of other *PME* genes as well, namely *Solyc03g123630 (PMEU1), Solyc07g064170 (PE1), Solyc07g064180 (PME2.1), Solyc06g051960 (LES.9028)* and *Solyc03g083360 (Les.10790)* (de Freitas et al. 2012b)*.* The increase in soluble Ca^2+^ concentration in the antisense plants is particularly noticeable in the apoplast and is associated with the lack of cell plasmolysis compared to control. Moreover, the pectin in the antisense plants was highly methylated compared to control. In sum, the role of free apoplastic Ca^2+^ concentration maintains proper Ca^2+^ homeostasis among different cellular compartments and prevents membrane leakage, hence reduced BER incidence (de Freitas et al. 2012b). In addition, PMEs are critical in regulating pectin composition which is directly influencing BER (de Freitas et al. 2012b). Even though numerous studies have correlated BER to Ca^2+^ homeostasis (Geraldson 1956; Spurr 1959; Adams and Ho 1993; Bar-Tal et al. 2001; de Freitas et al. 2011b; de Freitas et al. 2012b), findings from other studies suggest that aberrant Ca^2+^ homeostasis is a consequence and may not be the cause of BER (Nonami et al. 1995; Saure 2001; Rached et al. 2018; Matsumoto et al. 2021). For example, before and right after the onset of BER, the Ca^2+^ concentration is the same among all the fruits for the different tissue types (Nonami et al. 1995). As BER is developing further, the Ca^2+^ concentrations start to differ markedly. It is perhaps the organization of the pectin structure in the middle lamellae that is crucial to regulating the onset of BER in plants.

### Reactive oxygen species (ROS) and BER

Ca^2+^ and ROS signaling are both interrelated secondary messengers that respond to many environmental stresses. Ca^2+^ regulates ROS production, whereas ROS regulates Ca^2+^ homeostasis (Kobayashi et al. 2007; Jiang et al. 2011; Görlach et al. 2015). Whether ROS poses a threat to cells or has a role in response signaling depends on the equilibrium between ROS generation and detoxification (Sharma et al. 2012; Ayer et al. 2014). In plants, electron transport reactions in the plasma membrane (e.g. NADPH oxidase), the endoplasmic reticulum, the chloroplast and the mitochondria (e.g. the electron transport chain) are the major sources of ROS production (Trachootham et al. 2008). These sources produce free radicals such as superoxide anion, hydroxyl radicals as well as nonradical molecules like hydrogen peroxide and singlet oxygen (Sharma et al. 2012). Plant cells have evolved to alleviate the negative impacts of ROS by producing enzymatic and nonenzymatic antioxidants in the ROS scavenging pathway (Mittler 2002; Gratão et al. 2005). Enzymatic antioxidants consist of superoxide dismutase (SOD), ascorbate peroxidase (APX), monodehydroascorbate reductase (MDHAR), dehydroascorbate reductase (DHAR), glutathione reductase (GR), catalase (CAT), and others (Willekens et al. 1997; Trachootham et al. 2008; Marengo et al. 2016). The major nonenzymatic antioxidants include glutathione, ascorbate, as well as tocopherol, flavonoids, phenolic compounds, and carotenoids (Sies and Stahl 1995; Ayer et al. 2014). The Ascorbate-Glutathione pathway plays a significant role in detoxifying ROS in plants and consists of four main enzymes namely: APX, MDHAR, DHAR, and GR and two antioxidants: AsA and GSH (Noctor and Foyer 1998; Foyer and Noctor 2011).

Excessive ROS leading to lipid and protein oxidation, enzyme inhibition, and cell membrane leakage are all are associated with BER. Therefore, ROS is considered a critical component of BER onset and development (Dhindsa et al. 1981; Van Breusegem and Dat 2006; Sharma et al. 2012; de Freitas et al. 2018; Reitz and Mitcham 2021). Tomatoes grown under Ca^2+^-deficient conditions experience excess ROS accumulation and increased BER incidence that is associated with the upregulation of NADPH oxidase and SOD (Mestre et al. 2012). Similarly, peppers grown under saline conditions experience high ROS accumulation in the apoplast due to increased activity of NADPH oxidase activity (Aktas et al. 2005). On the other hand, many antioxidant genes such as *CAT*, *APX*, and *GR* are down-regulated in tomatoes grown under Ca^2+^ deficient conditions (Ming and Zhong-Guan 1995; Schmitz-Eiberger et al. 2002; Yang and Poovaiah 2002; Mestre et al. 2012). The tomato cultivar HM 4885, one of the preferred processing tomatoes in California, USA, experienced 85% BER incidence that was attributed to the down regulation of *CAT* leading to higher ROS accumulation (Reitz and Mitcham 2021). Consequently, the aberrant regulation of critical enzymes in the ROS detoxification pathway can lead to extensive H_2_O_2_ accumulation, lipid peroxidation and membrane breakdown, which subsequently results in increased BER incidence (Mestre et al. 2012).

Tomato varieties that have naturally high levels of ascorbate and antioxidants during the most sensitive stage of BER are more resistant to the disorder than those that have lower antioxidant levels, irrespective of the fruit Ca^2+^ concentration (Rached et al. 2018). Further, BER does not always consume the entire fruit (Fig. 2). This may be due to increased lignification, antioxidants, and oxidative stress-related proteins that inhibit further expansion of BER to the neighboring healthy tissues (Schmitz-Eiberger et al. 2002; Casado-Vela et al. 2005; Mestre et al. 2012; Reitz and Mitcham 2021).

Taken together, the ROS enzymes and antioxidants play a major role in BER development which is enhanced by insufficient Ca^2+^ concentration and abiotic stress (Noctor and Foyer 1998; Aloni et al. 2008; Rached et al. 2018). Specifically, the activation of enzymes in ROS production pathway as well as inhibition of enzymes in ROS scavenging pathway leads to membrane leakage and consequently higher BER incidence.

### Other physiological factors in BER development

Certain nutrients have antagonistic effects on the uptake of each other. High concentrations of monovalent cations in soils, such as potassium (K^+^), magnesium (Mg^+^), sodium (Na^+^) and ammonium (NH_4_^+^) have a negative impact on the uptake of divalent cation Ca^2+^, thereby increasing BER incidence (Taylor and Locascio 2004; Mengel and Kirkby 2012). For instance, a rise in NH_4_^+^ concentration in the nitrate/ammonium ratio suppressed the Ca^2+^ uptake and led to an increase in BER development (Geraldson 1956; Marti and Mills 1991; Nukaya et al. 1995; Bar-Tal et al. 2001; Taylor and Locascio 2004). The uptake of other elements such as boron (B^+^) may also influence BER incidence. Fruits that were collected from a BER-resistant accession showed a high correlation between B^+^ and Ca^2+^ concentration in the distal part of each fruit whereas the susceptible accession showed no correlation between the two elements (Watanabe et al. 2021). In this case, the link between B^+^ and Ca^2+^ might reveal a role in stabilizing the pectin structures in the cell wall.

Plant growth regulators also affect BER development. The plant growth regulators auxin and gibberellin (GA) are reported to accelerate fruit growth and cause an increase in BER (de Freitas et al. 2012a; Gaion et al. 2019). The decreased Ca^2+^ concentration that was observed in the fruits upon the GA application was attributed to increased activity of *Ca*^*2+*^*/H*^*+*^ *exchangers* and *Ca-ATPase* genes, that are responsible for Ca^2+^ transport into the storage organelles and the apoplastic space (de Freitas et al. 2012a). Specifically, GA application leads to the reduction of the apoplastic water-soluble Ca^2+^ content and enhanced cell membrane permeability (de Freitas, et al. 2012). Additionally, GA application leads to elevated ROS levels and decreased expression of many antioxidant genes such as APX, SOD, and CAT (Fath et al. 2001). On the other hand, application of growth retardants such as abscisic acid and Apogee (inhibitor of GA biosynthesis) to tomato plants showed reduced or no BER (de Freitas et al. 2011a; Barickman et al. 2014; de Freitas et al. 2014; de Freitas et al. 2018). Eliminating BER was attributed to the increased pericarp Ca^2+^ concentration and a higher number of functional xylem vessels in the placenta and pericarp tissues of fruits during the early growth stages (de Freitas et al. 2012a). These retardants also trigger antioxidant production to counter ROS activity, thereby further reducing BER incidence (de Freitas et al. 2018). Slower initial fruit growth rates are also associated with reduced BER incidence (Ho et al. 1987; Aktas et al. 2003; Aktas et al. 2005; Vinh et al. 2018; Watanabe et al. 2021). This suggests that the increased growth rate following pollination or after growth regulator application creates extensive stresses in the distal fruit part. This could lead to lower Ca^2+^ concentrations, and reduced cell wall stabilization and membrane integrity (Ikeda et al. 2017; Watanabe et al. 2021).

### Relationship between BER and fruit morphology

Fruit size and BER onset are positively correlated to one another in tomato (Marcelis and Ho 1999; Heuvelink and Körner 2001) and no study has reported the occurrence of BER in wild relatives and small fruited varieties of tomato (Ho and White 2005). As BER is only observed in cultivated plants, domestication may have driven BER as a consequence of selections for larger produce. The tomato gene *Cell Size Regulator* (*FW11.3*/*CSR)* increases fruit weight by increasing the cell size (Mu et al. 2017). *FW11.3* near isogenic lines (NILs) that carry the derived allele of *CSR* showed significantly higher BER incidence compared to *FW11.3* NILs that carry the wild type allele, indicating that *FW11.3*/*CSR* may have a role in BER development (Mu 2015). The association of BER with this fruit weight genes is likely indirect and not causative because many tomato varieties with the derived fruit weight alleles are resistant to BER.

In addition to fruit size, elongated fruit shapes are more prone to BER than the round-fruited varieties (Ku and Tanksley 1998; Ho and White 2005; Riboldi et al. 2018). Elongated fruit shape in tomato is controlled by only a handful of genes, namely *SUN, OVATE, OFP20* and *FS8.1* (Ku et al. 2000; Liu et al. 2002; Xiao et al. 2008; Sun et al. 2015; Wu et al. 2018). Among these genes, the round fruit allele of *fs8.1* is associated with low BER Incidence (Ku and Tanksley 1998). Moreover, the varieties San Marzano carrying the *OVATE* mutation and Banana Legs carrying the *SUN* mutation are highly susceptible to BER (Riboldi et al. 2018). Despite the demand for these produce shapes in the processing tomato industry, growers often avoid growing certain varieties due to potentially high yield losses. The likely mechanism of BER in elongated fruits has been proposed to be caused by the reduced functional xylem elements in the distal end of the fruit leading to reduced Ca^2+^ concentration compared to proximal end (Ho and White 2005; Riboldi et al. 2018).

### Genetic basis of BER

In addition to the physiological factors, tomato varieties display varying degrees of BER which suggests a genetic basis to the disorder (Adams and Ho 1992; Ho et al. 1995; Ho and White 2005). The earliest investigation in the genetic basis of BER came from studies using tomato introgression lines (ILs). These ILs consist of genomic segments of *Solanum pennellii* LA716 introgressed into *Solanum lycopersicum* cv M82 (Eshed and Zamir 1995). Among these lines, IL8–3 features lower BER Incidence compared to the M82 parent (Uozumi et al. 2012; Ikeda et al. 2017; Watanabe et al. 2021). This region was fine mapped to 610 kb corresponding 78 genes (Uozumi et al. 2012; Ikeda et al. 2017). Because the higher Ca^2+^ concentration in the distal part of the fruit and the initial slower growth rate in the BER resistant line, the results indicate that IL8–3 might harbor gene(s) affecting Ca^2+^ concentration and growth rate in the early stages of fruit development. Additionally, further use of these IL8–3 lines revelated that many Ca^2+^-transport-related genes such as cation exchanger (*CAX*), *Ca*^*2+*^*-ATPase*, *Ca*^*2+*^*-channel* and *Na*^*+*^*/Ca*^*2+*^ *exchanger* were differentially expressed between M82 and IL8–3 ten days after flowering but none of these genes mapped to location of IL8–3 on chr08 (Ikeda et al. 2016). These results may suggest that Ca^2+^-transport-related genes in other chromosomes are likely regulated by one of the 78 genes located in 610 kbp region in IL8–3. (Ikeda et al. 2017). Another IL, namely IL5–4, located on chr05 also featured differences in BER but in this case, the severity is higher in the IL than in the control M82 (Matsumoto et al. 2021). This locus has not been finemapped further.

**Fig. 3:**
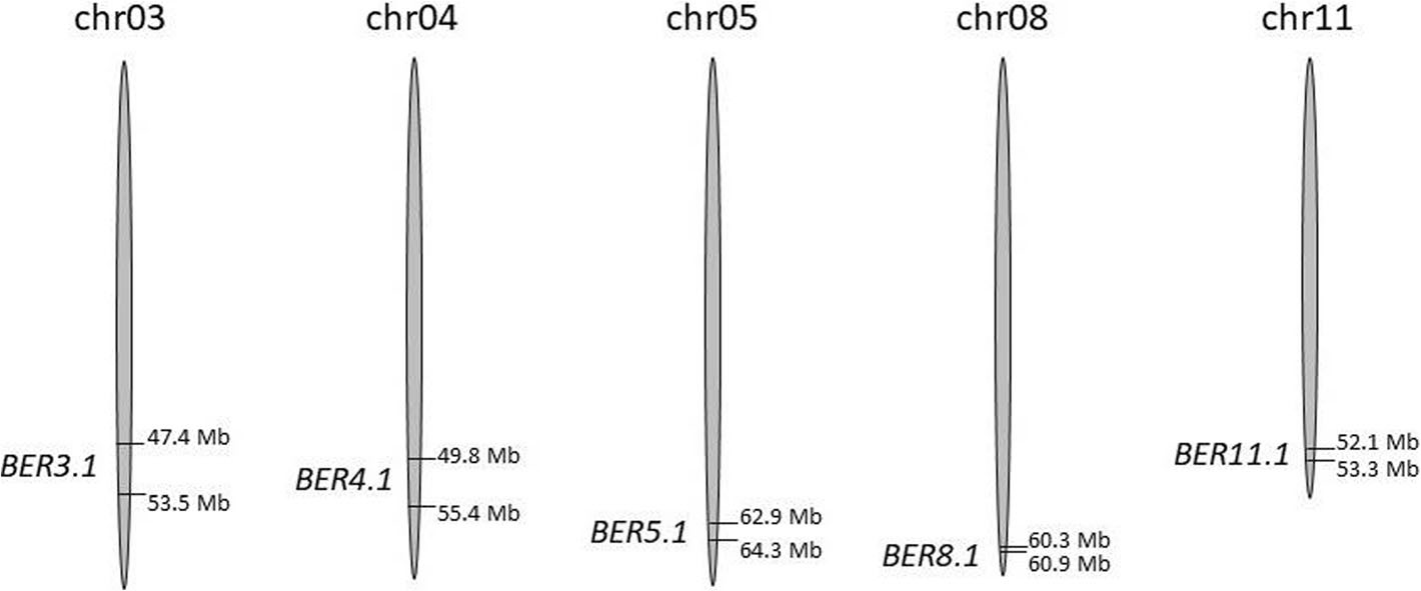
Location of the five BER loci in the tomato genome

Due to the low genetic diversity between closely related tomato accessions, the genetic basis of BER in populations derived from crosses among cultivars was hampered by the lack of molecular markers until recently. With the advent of the full genome sequence of tomato (Tomato Genome Consortium 2012), many resequencing projects enable the discovery of single nucleotide polymorphisms (SNPs) between closely related parents. Using the QTL seq approach, the enrichment of SNPs that are associated with the trait leads to the development of molecular markers to map BER loci in the population (Topcu et al. 2021). In populations derived from crosses between *Solanum lycopersicum var. cerasiforme* (*SLC*) and *S. lycopersicum var. lycopersicum* (*SLL*), four loci were identified: *BER3.1* and *BER3.2* on chr03, *BER4.1* on chr04 and *BER11.1* on chr11 (Topcu et al. 2021). *BER3.2* and *BER11.1* were further finemapped to 1.58 and 1.13 Mb respectively, whereas *BER11.1* was also mapped in another population derived from *SLL* cv Ailsa Craig and *SLL* cv Kentucky Beefsteak (Prinzenberg et al. 2021). The studies showed that *BER3.2* is likely corresponding to the fruit weight gene *FW3.2/KLUH* which was segregating in one of the populations (Topcu et al. 2021) as larger fruit tend to be more susceptible to BER than smaller fruits (see above section). In sum, the studies into the genetic basis of BER identified a total of five loci in tomato namely: chr 03, chr 04, chr 05, chr 08 and chr 11 and excluding *FW3.2/KLUH* (Fig. 3). The cloning of the genes in these loci should provide novel insights into the onset and early developmental stages of BER.

## Conclusion and future perspectives

The research on BER has led to the findings that the interplay of Ca^2+^ homeostasis and ROS accumulation perform critical roles in the development of the disorder. Together, they affect membrane stability and cell wall properties as to the degree of pectin methylation and hence BER appearance. Because the combination of environmental stress and nutritional factors affect the incidence of BER greatly, this disorder is often difficult to manage in field and greenhouse growth conditions in many agricultural settings. Going forward, growers will need to remain vigilant and pursue proper field management practices such as mulching, effective water drainage, proper irrigation systems, balanced fertilizer applications, and soil reclamation, which is the removal of salt from the root zone (Machado and Serralheiro 2017; Hagassou et al. 2019). Other management strategies such as the use of growth retardants can also help alleviate BER symptoms, but these are only available to commercial growers. On the other hand, a stronger emphasis on harnessing the power of the genetic variation in crop germplasm to at least reduce BER is critical. For example, a focus on the increased production of antioxidants in breeding programs should ameliorate the incidence and severity of BER. These high antioxidant-producing accessions would prevent lipid and protein oxidation, membrane breakdown, cell plasmolysis and hence BER. Another focus in breeding programs should be on vegetable varieties that feature a slower growth rate following pollination to avoid developing BER. As the genetic studies start to shed light on the causal genes underlying BER, new solutions to crop improvement in many vegetables are possible. For example, down regulation or knock outs of BER susceptibility genes using CRISPR-Cas gene and/or promoter editing should lead to the development of more resistant commercially produced varieties. Therefore, the toolkit to improve BER is expected to expand with new means for breeders to develop varieties that are more resistant to this often-devastating physiological disorder.

## Data Availability

Not applicable.

## Abbreviations

ABA: Abscisic acid
NH_4_^+^: Ammonium
APX: Ascorbate peroxidase
BER: Blossom-end rot
B^+^: Boron
Ca^2+^: Calcium
CAT: Catalase
*CAX*: Cation exchanger
*CSR*: Cell Size Regulator
DHAR: Dehydroascorbate reductase
GA: Gibberellic acid
GR: Glutathione reductase
ILs: Introgression lines
Mg^+^: Magnesium
MDHAR: Monodehydroascorbate reductase
NILs: Near isogenic lines
PMEs: Pectin methylesterases
K^+^: Potassium
ROS: Reactive Oxygen Species
*SLL*: *S. lycopersicum var. lycopersicum*
SNPs: Single nucleotide polymorphisms
Na^+^: Sodium
*SLC*: *Solanum lycopersicum var. cerasiforme*
SOD: Superoxide dismutase
